# High-grade uterine corpus-confined endometrial cancer with lymphadenectomy: does adjuvant therapy improve survival?

**DOI:** 10.4274/tjod.galenos.2019.04578

**Published:** 2019-10-10

**Authors:** Çiğdem Kılıç, Caner Çakır, Dilek Yüksel, Yasin Durmuş, Nurettin Boran, Günsu Kimyon Cömert, Alper Karalök, Gökhan Boyraz, Taner Turan

**Affiliations:** 1 University of Health Sciences, Etlik Zübeyde Hanım Women’s Diseases Training and Research Hospital, Clinic of Gynecologic Oncology, Ankara, Turkey

**Keywords:** Adjuvant therapy, endometrial cancer, high risk

## Abstract

**Objective:**

To evaluate the necessity of adjuvant therapy and other prognostic factors in high-grade uterine corpus-confined endometrial cancer (EC) with lymphadenectomy performed.

**Materials and Methods:**

This study included 120 patients who had endometrioid-type grade 3, serous-type, clear cell-type, and undifferentiated-type EC and underwent lymphadenectomy.

**Results:**

Patients with high-grade uterine corpus-confined EC who underwent lymphadenectomy were evaluated. The modality of adjuvant therapy performed was not a predictor for the site of recurrence. The loco-regional recurrence rate decreased from 9.5% to 3.8% in patients who received radiotherapy. However, this difference was not statistically significant (p=0.206). In addition, performing adjuvant chemotherapy did not alter the risk of extrapelvic recurrence. Only International Federation of Gynecology and Obstetrics 2009 stage was significant in the univariate analysis. On the other hand, age, tumor type, number of removed lymph nodes, presence of myometrial and lymphovascular space invasion, tumor size and adjuvant therapy modality were not related with disease-free survival.

**Conclusion:**

Performing adjuvant therapy and therapy modality does not improve oncologic outcomes in intermediate and high-risk patients. However, radiotherapy reduced the risk of local recurrence by more than 50%. Vaginal brachytherapy was efficient as external beam radiotherapy. Therefore, vaginal brachytherapy should be used for these patients in order to reduce loco-regional recurrence even if it is not reported to be effective on disease-free survival.

**PRECIS:** In this study, we aimed to evaluate the use of adjuvant therapy in patients with high-grade uterine corpus-confined endometrial cancer who underwent lymphadenectomy.

## Introduction

Endometrial cancer (EC) is the most frequent cancer of the female genital tract and the fourth cancer among all cancer types^(1)^. According to GLOBOCAN 2012 data, 320.000 new cases are diagnosed each year^(2)^. EC is mostly diagnosed at the early stage and the main treatment is surgery^(3)^. Five-year overall survival (OS) is over 80% for low-grade tumors in early-stage EC^(4)^. Surgery consisting of total hysterectomy + bilateral salpingo-oophorectomy and evaluation of the extent of the disease is the standard initial therapy. EC has been staged surgically according to the International Federation of Gynecology and Obstetrics (FIGO) since 1988^(5)^. FIGO revised the staging system in 2009^(6)^.

The use of adjuvant therapy in uterine corpus-confined EC is controversial. Reports revealed that external beam radiotherapy (EBRT) decreased loco-regional recurrence in patients with deep myometrial invasion, tumor with poor differentiation, and advanced age, but EBRT did not improve OS^(7,8)^. Other trials that investigated the difference between adjuvant therapy modalities revealed that EBRT had serious adverse effects. Vaginal brachytherapy (VBT) could be a type of adjuvant radiotherapy (RT) given in patients with EC because of its tolerability^(9,10)^. This study was designed to evaluate the necessity of adjuvant therapy and other prognostic factors in patients with high-grade uterine corpus-confined EC who underwent lymphadenectomy.

## Materials and Methods

This study included 120 patients whose staging surgeries (total hysterectomy and bilateral salpingo-oophorectomy and pelvic and paraaortic lymphadenectomy) were performed in our oncology clinic between January 1993 and December 2017 and who had uterine corpus- confined endometrioid-type grade 3, serous-type, clear cell-type, and undifferentiated-type EC according to the final pathology results. Data of the patients were obtained from the hospital’s electronic database, and the patients’ files and pathology results were analyzed, retrospectively. Patients whose surgeries had not been performed in our clinic, with endometrioid-type grade 1 and 2 or mixed-type adenocarcinoma, whose tumors had a sarcoma component, with synchronized primary tumor, whose surgeries had not included lymphadenectomy, who were lost during follow-up, who died in the first month after surgery, and those who underwent neo-adjuvant treatment were excluded. Ethical board approval exists for this study. Staging was performed according to the FIGO 2009 criteria. Tumor size was measured as the longest tumor diameter in the uterine corpus after fixation in a paraffin block. Lymphovascular space invasion (LVSI) was defined as the tumoral cells or cell clusters held on vessel walls that were stained with hematoxylin and eosin in the pathologic sections, containing both tumor and the surrounding healthy tissue. The omentum was pathologically examined through 2-3 sections taken from the macroscopic tumor and suspicious areas, or through 3-5 sections taken from healthy looking omentum tissue. Pathologic examinations of the hysterectomy material were performed with at least 4 cut-out sections. Lymph node examinations were performed as follows: the material was embedded in a paraffin block (i) directly, if the size was less than 1 cm; (ii) with  horizontally cutting at least into two pieces according to size, if it was more than 1 cm. In the presence of the macroscopic tumor, only that part was directly taken into the paraffin block. The sections were evaluated after hematoxylin and eosin staining. Standard staging surgery included cytologic sampling, total abdominal hysterectomy, bilateral salpingo-oophorectomy, systematic pelvic and paraaortic lymphadenectomy, and omentectomy. During the intra-operative observation, cytoreductive surgical techniques were performed in addition to staging surgery in the presence of a macroscopic tumor. Lymphadenectomy was performed in most patients by skeletonizing the pelvic and paraaortic regions. Bilateral pelvic lymphadenectomy was performed to complete skeletonization with all lymphatic tissue of the common, external, and internal iliac vessels, and the obturator fossa, which was removed after visualization of the obturator nerve. The superior surgical dissection margin for the pelvic nodes was the aortic bifurcation, and the anterior distal surgical dissection margin was the circumflex iliac vein. Presacral lymphatic tissue was removed separately. The upper limit of the paraaortic lymphadenectomy was the left renal vein. All lymphatic tissue was then removed from the precaval, laterocaval, interaortacaval, preaortic and lateroaortic regions up to the left renal vein. All surgeries were performed using open surgical techniques, and pathologic findings were examined and interpreted at a single institution. The use and type of adjuvant therapy was decided by a gynecologic oncology council and senior surgeons. Adjuvant RT was administered as EBRT and/or VBT. Low-dose cisplatin used within concurrent chemoradiotherapy was not accepted as systemic therapy due to it being non-curative. Recurrence following surgery used as the initial therapy for a period of one month or progression during adjuvant therapy was regarded as refractory disease. One month after the completion of adjuvant therapy, a follow-up examination was performed and the non-appearance of disease had to be documented. From this point, any abnormal finding was evaluated as recurrent disease. Loco-regional recurrence was defined as relapses located in the vagina, vaginal vault, and pelvic side wall below the level of the linea terminalis. The recurrence region between the level of the linea terminalis and diaphragm was called “upper abdominal” and all other regions were called extra-abdominal. Recurrence in the liver parenchyma and bone was accepted as extra-abdominal; ascites proven with cytologic evaluation and peritoneal carcinomatosis was accepted as upper abdominal. Recurrence was defined after the evaluation of the patient’s clinical, radiologic, and pathologic findings by performing pelvic and systematic examinations, abdominal X-ray, abdominopelvic and thoracic computed tomography (CT) or magnetic resonance imaging. The decision of recurrence-related therapy was made by a gynecologic oncology council. The patients were followed-up quarterly in the first two years, semi-annually up to five years, and annually thereafter. Pelvic examination, abdominopelvic ultrasonography, complete blood count, and blood chemistry were performed. Chest X-ray was performed yearly unless there was clinical suspicion. Thoracic and/or abdominal CT was used when needed. Canser antigen 125 levels were used in the follow-up, even though they were not used routinely. The time period from initial surgery to recurrence or the last visit was accepted as disease-free survival (DFS), and the time period from the initial surgery to disease-related death or the last visit was accepted as disease-specific survival (DSS). Time to recurrence (TTR) was defined as the period of time from the initial surgery to relapse in patients with recurrence. Categorical variables were analyzed using Kaplan-Meier survival analysis using the log-rank test to determine whether they had statistically significant effects on DFS or DSS.

### Statistical Analysis

Whether the continuous and discrete numeric variables had statistically significant effects were calculated using univariate Cox proportional hazard regression analysis. Multivariate backward stepwise Cox proportional hazard regression analysis was used to determine the effects of variables effective on survival after the univariate statistical analysis. Factors with a p value of <0.25 in univariate analyses were included as candidate variables in multivariate analyses. P values <0.05 were considered statistically significant for the results. Data analyses were performed using the SPSS for Windows 11.5 package program.

## Results

**Figure 1:**
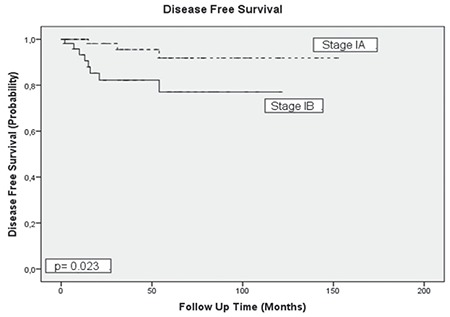
Relationship between disease free survival and stage

The mean age of the patients was 60 (range, 38-79) years. The tumor type was grade 3 endometrioid in 76 patients, clear cell in 24, serous in 18, and undifferentiated in two. Sixty-seven (55.8%) patients were stage 1A and 53 (44.2%) patients were stage 1B according to the FIGO 2009 criteria. Myometrial invasion was not detected in 18 patients. The median tumor size was 35 (range, 5-150) mm. The median number of removed lymph nodes was 51 (range, 3-118). Lymphadenectomy was performed with ≥21 lymph nodes in 91% of the patients. LVSI was positive in 38 patients, cervical glandular invasion was positive in four, and peritoneal cytology was positive in one patient. Data related to surgico-pathologic factors are summarized in Table 1. Adjuvant therapy was performed in 90 (75%) of the patients. The most frequent adjuvant therapy was RT and 78 (65%) patients received RT with/without chemotherapy. Thirty-six (30%) patients received VBT only, 28 (23.3%) patients received EBRT only, and five (4.2%) patients received VBT + EBRT. Information about the type of RT could not be found in nine patients’ files. Adjuvant systemic therapy was applied to 21 (17.5%) patients, 12 (10%) of whom received only chemotherapy. Data related to adjuvant therapy are shown in Table 2. Tumor type was a significant predictor for determining the modality of adjuvant therapy. Adjuvant RT rates were 73% in patients with grade 3 endometrioid-type tumors and 50% in patients with non-endometrioid-type tumors (p=0.009). Similar rates were found for systemic therapy between the same groups of patients. Chemotherapy was performed in 6.6% of patients in the endometrioid group and 36.4% of patients in the non-endometrioid group (p<0.001). In spite of this, tumor type, FIGO 2009 stage, and presence of myometrial invasion did not determine the adjuvant therapy modality in patients receiving RT only (p=0.068, p=0.883, and p=0.504, respectively). The modality of adjuvant therapy performed was not a predictor for the site of recurrence. The loco-regional recurrence rate decreased from 9.5% to 3.8% in patients who received RT (VBT and/or EBRT with/without chemotherapy). However, this difference was not statistically significant (p=0.206). In addition, performing adjuvant chemotherapy did not alter the risk of extrapelvic recurrence. The extrapelvic recurrence rates were 4.8% and 6.1% in the chemotherapy group and non-chemotherapy group, respectively (p=0.818). The median follow-up period was 33 (range, 2-152) months. It was observed that during this period, 11 (9.2%) patients had recurrence and three (2.5%) patients died of the disease. In the entire cohort, none of the patients had refractory disease. The median TTR was 15 (range, 2-54) months in patients who developed recurrence. Four (3.3%) patients had recurrence only in the pelvic region and seven (5.8%) patients had extrapelvic recurrence; six (5%) of which were in extra-abdominal regions (Table 3). In our study, the 5-year DFS was 87% and the 5-year DSS was 97%. The factors affecting the prognosis were determined by using DFS because there were only three disease-related deaths. Accordingly, only the FIGO 2009 stage was significant in the univariate analysis. The 5-year DFS was 92% in stage 1A and 81% in stage 1B (p=0.023) (Figure 1). On the other hand, age, tumor type, number of removed lymph nodes, presence of myometrial and LVSI , tumor size, and adjuvant therapy modality were not related with DFS (Table 4). Stage (2009 FIGO stage 1A vs. 1B), presence of myometrial invasion (noninvasive vs. myoinvasive), LVSI (negative vs. positive), and adjuvant RT type (VBT vs. EBRT ± VBT) whose p values were found below 0.25 on univariate analysis, were evaluated using multivariate analysis. However, a model could not be developed because of the correlation within these factors. Also, a multivariate analysis defining recurrence risk could not be obtained. The efficacy of prognostic factors was assessed through subgroup analysis in patients with stage 1B disease (n=53). The median follow-up period of this group was 36 (range, 2-121) months. In the follow-up, eight (15.1%) patients had recurrence and three (5.7%) patients died of the disease. It was considered that prognostic factors were ineffective for determining DFS using univariate analysis. Age (≤60 year vs. >60 year; p=0.522), tumor type (endometrioid vs. non-endometrioid; p=0.377), number of removed lymph nodes (≤48 vs. >48; p=0.072), LVSI (negative vs. positive; p=0.507), tumor size (≤40 mm vs. >40 mm; p=0.671), adjuvant therapy (received vs. not received; p=0.457), adjuvant RT (received vs. not received; p=0.693), type of adjuvant RT (VBT vs. EBRT ± VBT; p=0.114), adjuvant chemotherapy (received vs. not received; p=0.869), and RT+ chemotherapy therapy (received vs. not received; p=0.858) showed no statistical significance.

## Discussion

This study suggested that clinical, surgical, and pathologic factors, except for stage, had no prognostic value in high-grade uterine corpus-confined EC with lymphadenectomy performed. The 5-year DFS decreased from 92% to 81% in patients with deep myometrial invasion (stage 1B). The entire cohort of patients had recurrence, 63% (n=7/11) in the extrapelvic region and 55% (n=6/11) in the abdominal region. However, local (RT) or systemic (chemotherapy) therapy had no beneficial effect or did not change the recurrence site. Despite that, RT decreased pelvic recurrence rates from 9.5% to 3.8% with no statistical significance. In addition, the type of RT had no effect on oncologic outcomes. There have been opposing studies in the literature offering the utility of adjuvant therapy and discussing the modality types of the therapy. Gupta et al.^(11)^ evaluated 33.600 patients by using the National Cancer Database to examine the impact of adjuvant radiation therapy on OS in patients with high-intermediate risk stage 1 EC. They accepted stage 1B and/or grade 3 patients as the high-intermediate risk group. Approximately three-quarters of the patients underwent lymphadenectomy. The average number of removed lymph nodes was not obvious. The study showed a statistically significant difference in OS rates between the surgery alone vs. surgery + adjuvant RT groups. According to this study, loco-regional control with adjuvant RT causes an improvement in 5-year OS (respectively, 79.2% vs. 83.3%, p<0.0001)^(11)^. Postoperative Radiation Therapy in Endometrial Carcinoma (PORTEC-1) was a study that included patients with grade 1 EC and ≥50% invasion, grade 2 with any invasion, or grade 3 with <50% invasion. In this study, 715 patients were randomized to the surgery alone vs. surgery + EBRT arms. Surgery was performed without lymphadenectomy. This study suggested that postoperative radiation therapy in stage 1 EC decreased loco-regional recurrence rates, but did not change OS (85% vs. 81%, p=0.31). For the prevention of loco-regional recurrence (5% vs. 18%), radiation therapy should be used for patients with high-intermediate risk who have two of these factors; age ≥60 years, grade 3 and deep myometrial invasion^(7)^. After 15 years of follow-up, 426 patients from the PORTEC-1 trial were re-evaluated. Loco-regional recurrence rates were 6% for EBRT vs. 15.5% for the surgery alone group (p<0.0001). The 15-year OS was 52% vs. 60%, and the failure-free survival was 50% vs. 54%. These rates showed no statistical significance^(12)^. The Gynecologic Oncology Group 99 trial^(8) ^was designed to determine the effect of adjunctive whole pelvic radiation therapy (EBRT) on loco-regional recurrence and OS rates. The entire cohort consisting 447 patients with FIGO stage IB, IC and II disease with intermediate risk factors were accepted as the high-intermediate and low-intermediate risk groups. High-intermediate risk factors were defined as moderate, poorly differentiated tumor, presence of lymphovascular invasion, outer third myometrial invasion, age 50 years or older with any two risk factors or 70 years or older with any of the risk factors. All patients underwent lymphadenectomy. For patients in the low-intermediate risk group, adjuvant RT was not recommended. After 2 years of follow-up, no additional therapy group had an estimated cumulative incidence of recurrence rate of 12%, and the RT group had 3% (p=0.007). The OS rates showed no statistically significant difference (p=0.557). This study suggested that additional RT in uterine corpus-confined EC should be given to patients with high-intermediate risk factors. In the PORTEC-2 trial, 427 patients with stage 1 or 2A disease who had high-intermediate risk factors and underwent EBRT or VBT were compared for recurrence, survival, and toxicity. High-intermediate risk factors include age more than 60 years, FIGO 1988 stage 1C grade 1 or 2 disease, or stage 1B grade 3 disease and stage 2A disease at any age. However, routine lymphadenectomy was not performed; only suspicious lymph nodes were removed. The 5-year loco-regional recurrence rates were 2.1% for the EBRT group and 5.1% for the VBT group (p=0.17). No difference was found in OS (respectively, 79.6% vs. 84.8%, p=0.57) and disease free survival (respectively, 78.1% vs. 82.7%, p=0.74) rates. Grade 1-2 gastrointestinal toxicity was lower in the VBT group than in the EBRT group (12.6% vs. 53.8%) at the completion of adjuvant therapy. However, after 2-years of follow-up, the difference between the reported toxic effects decreased and showed no statistical significance. In this study, it was suggested that VBT should be the choice of treatment as adjuvant therapy because of the gastrointestinal adverse effects^(9)^. PORTEC-3 was a multicenter, open-label, randomized, international trial investigating the survival rates and adverse effects of adjuvant therapy modalities in patients with EC^(10)^. Women with high-risk EC were randomized to radiation therapy alone or concurrent chemoradiotherapy arms to evaluate the difference between the two adjuvant therapy modalities. Lymphadenectomy was not performed for all patients. The 5-year OS was 81% in the chemoradiotherapy group vs. 76% in the RT group (p=0.11). The 5-year DFS was 75% vs. 68%, respectively (p=0.022). Grade 2 or higher sensory neuropathy was found to have a statistically significant difference between the two groups at 36 months (8% vs. 1%, respectively, p<0.0001). For patients with stage 1 and 2 disease, chemoradiotherapy did not improve OS and should not be recommended as a standard procedure.

### Study Limitations

The retrospective nature of the study is its most important limitation. The small sample size of the study group is another disadvantage. However, the entire cohort consists of patients who underwent lymphadenectomy. The median number of removed lymph nodes was 51, and 90% of patients had 21 or more lymph nodes removed. This allowed us to create a study group consisting of uterine corpus-confined disease in which nodal spread was common. Thus, a homogenized cohort was obtained. This is the most remarkable advantage of this study. In addition, the other inclusion and exclusion criteria strengthened the study homogenization.

## Conclusion

Performing adjuvant therapy and therapy modality do not improve oncologic outcomes in patients at intermediate and high risk. However, RT reduced the local recurrence risk by more than 50%. VBT was efficient as EBRT. Therefore, VBT should be used for these patients in order to reduce loco-regional recurrence, even if it is not reported to be effective on DFS. For more accurate results, more randomized controlled trails should be performed in patients with uterine corpus-confined EC who have undergone systematic lymphadenectomy.

## Figures and Tables

**Table 1:**
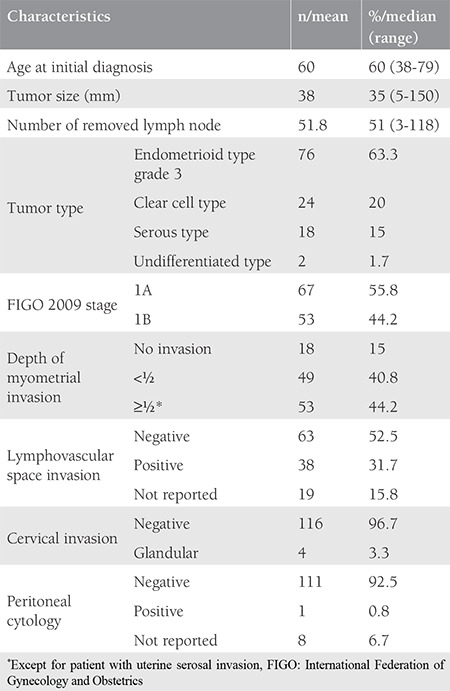
Clinical, surgical and pathological characteristics of patients

**Table 2:**
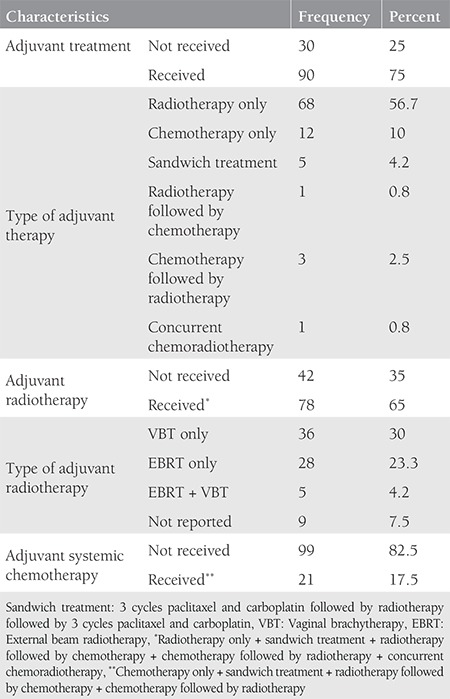
Adjuvant treatment

**Table 3:**
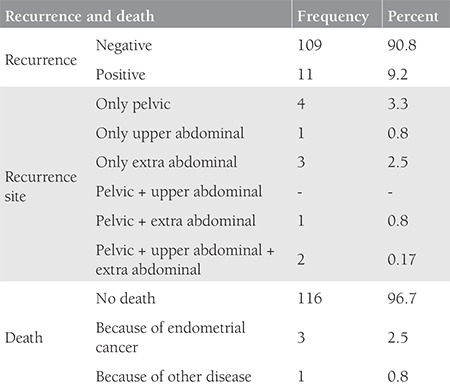
Recurrence, recurrence site and death

**Table 4:**
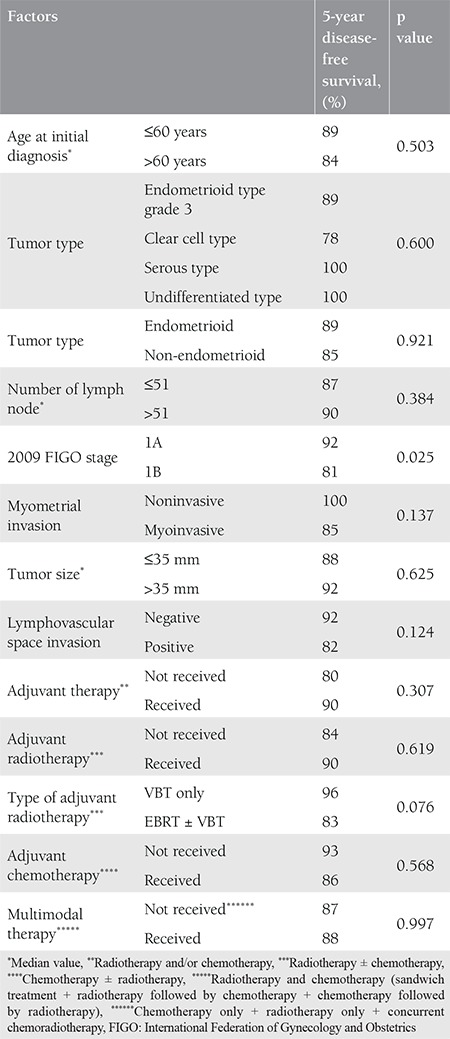
The factors predicting disease-free survival, univariate analysis
